# Translaminar circuits formed by the pyramidal cells in the superficial layers of cat visual cortex

**DOI:** 10.1007/s00429-017-1588-7

**Published:** 2017-12-12

**Authors:** German Koestinger, Kevan A. C. Martin, Elisha S. Rusch

**Affiliations:** Institute of Neuroinformatics, UZH/ETH, Winterthurerstrasse 190, 8057 Zurich, Switzerland

**Keywords:** Visual cortex, Pyramidal neuron, Layer 5, Dendrite, Synapse, Postsynaptic target

## Abstract

Pyramidal cells in the superficial layers of the neocortex provide a major excitatory projection to layer 5, which contains the pyramidal cells that project to subcortical motor-related targets. Both structurally and functionally rather little is known about this interlaminar pathway, especially in higher mammals. Here, we made sparse ultrastructural reconstructions of the projection to layer 5 of three pyramidal neurons from layer 3 in cat V1 whose morphology, physiology, and synaptic connections with layers 2 and 3 were known. The dominant targets of the 74 identified synapses in layer 5 were the dendritic spines of pyramidal cells. The fractions of target spiny dendrites were 59, 61, and 84% for the three cells, with the remaining targets being dendrites of smooth neurons. These fractions were similar to the distribution of targets of unlabeled asymmetric synapses in the surrounding neuropil. Serial section reconstructions revealed that the target dendrites were heterogenous in morphology, indicating that different cell types are innervated. This new evidence indicates that the descending projection from the superficial layer pyramidal cells does not simply drive the output pyramidal cells that project to cortical and subcortical targets, but participates in the complex circuitry of the deep cortical layers.

## Introduction

The neocortex is a three dimensional structure whose most prominent feature is its lamination, which is created by different cell types packed in different densities. These layers are linked by a network of interlaminar connections that have been the basis of the major theories of cortical processing (Hubel and Wiesel 1962; Gilbert and Wiesel 1983; Douglas and Martin 1991; Bastos et al. 2012). The superficial layers of the visual cortex of higher mammals also support a strongly recurrent local circuit (Binzegger et al. 2004) where the pyramidal cells provide a massive input to each other and to smooth inhibitory neurons, whose main targets are the superficial pyramidal cells (Koestinger et al. 2017). The same pyramidal cells send their output to the deep layers where they provide an estimated 60% of the excitatory synapses of layer 5 pyramidal cells (Binzegger et al. 2004). In the reciprocal projection, the layer 5 pyramidal cells provide a much smaller fraction (15%) of the total number of excitatory synaptic inputs of the superficial layer pyramidal cells (Binzegger et al. 2004).

The axons of superficial layer pyramidal cells in higher mammals branch to form multiple clusters of boutons (Martin and Whitteridge 1984a) (Kisvarday et al. 1986) (Binzegger et al. 2007) (Martin et al. 2017). Many such pyramidal cells collectively form a structure called the cortical ‘daisy’, which is ubiquitous in the neocortex of higher mammals (Douglas and Martin 2004). The center of the daisy is formed by the primary cluster of boutons (termed the ‘local’ cluster) around the dendritic tree of the parent cell and this local cluster contains the largest number of boutons of any cluster. The main axon has radial ‘spokes’ that form additional clusters of boutons in the superficial and deep layers. These distal clusters vary in number, but the number of boutons in each cluster is not constant but diminishes exponentially across all successive clusters (Binzegger et al. 2004; Martin et al. 2017). In the axonal projection to the deep layers, one cluster typically forms radially beneath the soma of the parent cell. Lateral clusters do occasionally form in layer 5 and they have a spacing similar to that of the distal clusters in the superficial layers (Kisvarday et al. 1987).

In the visual cortex, one common explanation for the daisy is that it is responsible for physiological properties like co-linear facilitation and cross-orientation inhibition. These hypotheses predict that clear differences should be found in the fraction of smooth cells (GABAergic, inhibitory cells) in the various clusters (Martin 1988). In our recent investigation of the cat’s visual cortex we did indeed find large differences in the proportion of smooth and spiny (glutamatergic, excitatory cells) that were postsynaptic targets of the local and distal bouton clusters (Koestinger et al. 2017). The difficulty for these hypotheses, however, was that the variance we observed in the fraction of target smooth cells did not correlate with the similarity or difference of the orientation domain of the cluster and the orientation preference of the parent cell. We also found that synapses in all clusters were similar in size, suggesting that the synaptic strengths were similar regardless of whether the synapses were formed in orientation domains of similar or different preferences to that of the parent cell. These observations suggest that the heterogeneity of targets found in the daisy clusters reflects a need to provide contextual information to each neuron in the superficial layers.

Given the strength of the descending projection from pyramidal cells in the superficial layers, it is unsurprising that the receptive fields of layer 5 cells bear a strong resemblance to those of the superficial layer pyramidal neurons lying radially above them (Hubel and Wiesel 1962; Gilbert 1977). Indeed, this radial organization of the interlaminar connections is one design feature that seems necessary to create the common properties defining functional columns. This interlaminar projection is, however, a canonical feature of neocortex and is not a unique feature of cortical areas that have functional columns (Douglas and Martin 2004). Thus, while this translaminar projection is probably necessary for columns, it is not sufficient to explain why the functional architecture is expressed as columns in higher mammals.

Virtually all the superficial layer pyramidal cells have axons that project through the white matter to other cortical areas. As the main axon projects radially into the white matter, it forms a collateral arborisation in layer 5. The layer 5 pyramidal cells are the only cells that project to motor-related structures, like the superior colliculus and pons in the case of V1, thus the nature of their input is significant in understanding the how this key component of the cortical output is created. Binzegger et al. (2004) calculated that about 60% of the excitatory synapses on the dendrites of layer 5 pyramidal cells originate from superficial layer pyramidal cells. Other inputs to layer 5 neurons in the cat include intralaminar connections from other layer 5 neurons (Gabbott et al. 1987), thalamus (Cunningham and Levay 1986; Anderson et al. 2009), and corticocortical connections (Hubener et al. 1990; Bullier et al. 1984).

Given its significance, surprisingly little is known about the targets of the descending projection. In an in vitro study of rat and cat cortex, Thomson et al. (2002) commented anecdotally on two instances of synaptic connections between a layer 3 pyramidal cell and a large layer 5 pyramidal cell in cat V1, which ‘were reminiscent of similar connections in rat cortex’, i.e. had depressing EPSPs. In their structural study of the connection Kisvarday et al. (1986) reported on the axons of two layer 3 pyramidal cells in cat V1 where 95% of the synapses in all the bouton clusters (sampled in both superficial and deep layers) were formed with spiny cells, the remainder with GABAergic smooth dendrites. This small variation in the ratio of spiny vs. smooth targets is very different from what we found in our more recent structure–function study of pyramidal cells in the cat where we found that the proportion of spiny neurons varied from 50 to 100% of the targets of the boutons (Koestinger et al. 2017). We further demonstrated that these proportions of smooth and spiny dendritic targets bore no relation to the degree of similarity of the orientation domain of the parent cell and that of the cluster domain sampled for EM.

In the present study, we extended the analyses of Koestinger et al. (2017) using three of the same pyramidal cells to analyse the synapses formed by the bouton clusters in the deep layers. Serial section electron microscopic reconstructions revealed a heterogeneity postsynaptic dendrites originating from both smooth and spiny neurons, indicating that the descending projection does not simply drive the layer 5 output cells, but participates in a complex circuitry in the deep layers.

## Results

**Fig. 1:**
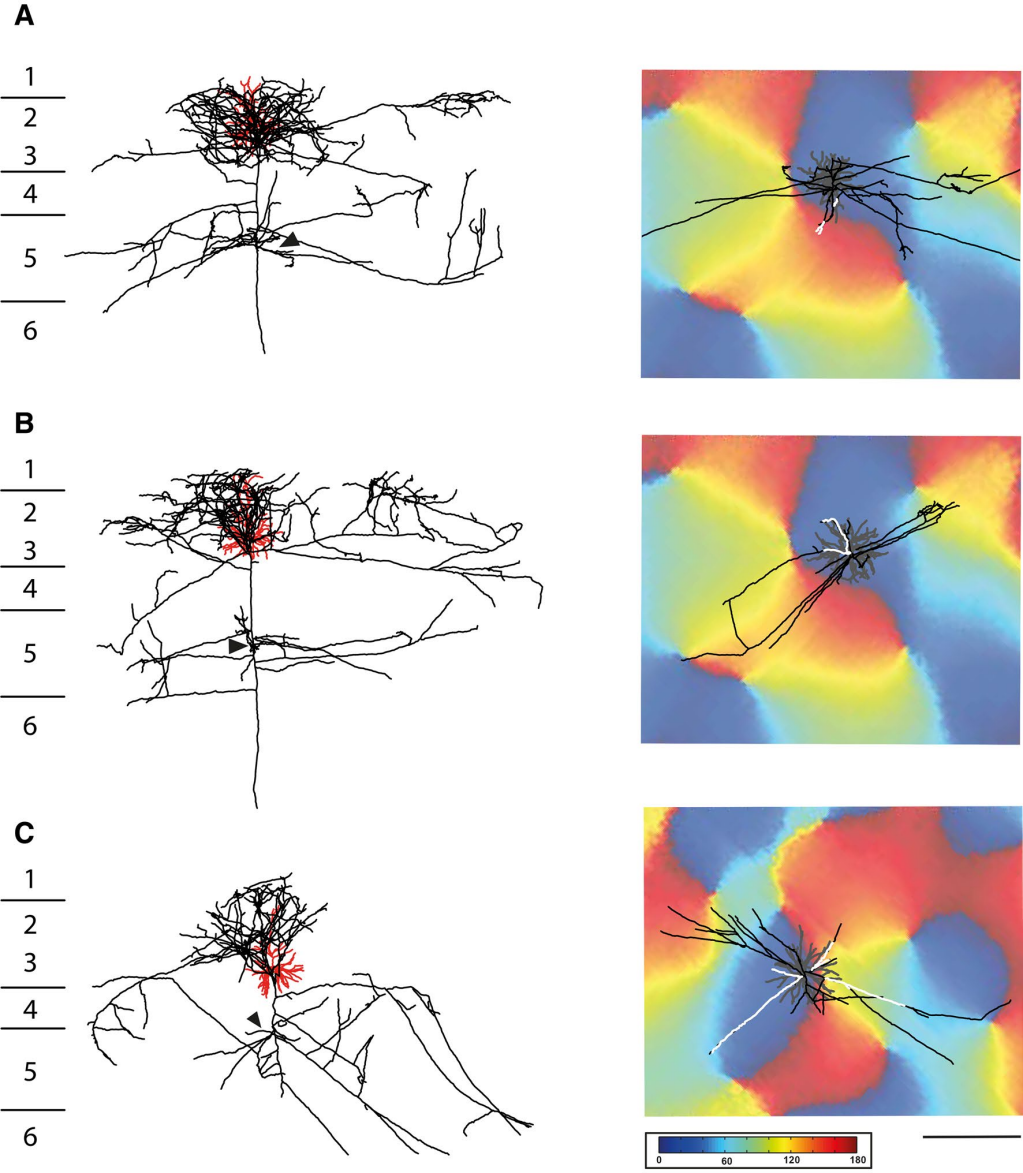
3D reconstruction of three pyramidal cells (black axon and red dendrite) shown in coronal view in the left column (layer boundaries depicted with numbers on the left). Black arrowheads indicate the region of axons further examined at electron microscopic level. The right hand column shows the orientation maps obtained by optical imaging for each neuron, as well as the view from the white matter of the deep layer axon (black, with segment reconstructed at EM in white) and the soma and dendritic tree of origin (grey). Scale bar 0.5 mm. Colour code for orientation map: 0 deg., horizontal, 90 deg., vertical

The data were obtained from three pyramidal neurons from layer 3 in cat primary visual cortex V1 (Fig. 1). The cells were characterized physiologically in vivo and then filled intracellularly with horseradish peroxidase (HRP). The organization of the superficial clusters of these and other pyramidal cells were previously studied in relation to the orientation map and the targets of their axons were identified by electron microscopy (Koestinger et al. 2017; Martin et al. 2017, 2014b). Here, we compared directly the targets and ultrastructural features of the bouton clusters formed in the deep layers with those of clusters formed in superficial layers.

Light microscopic reconstructions of the 3 pyramidal cells in Fig. 1 show (left) the characteristic pyramidal dendritic tree (red), enveloped with the black local axon cluster, with linear segments projecting laterally to form smaller distal clusters. The main axon descends, forming very few boutons on its passage through layer 4, but the axon typically forms a cluster in layer 5 radially beneath the soma and occasionally additional clusters laterally in layer 5. The arrowheads indicate the region where the sample axon segments were taken for ultrathin serial sectioning. The main axon then enters the white matter. The right column of Fig. 1 shows the associated orientation maps obtained by optical imaging of the intrinsic signal in the superficial layers. Superimposed on the maps are tangential representations of the dendritic tree (grey) and the portion of the axon in layer 5. The reconstruction has been rotated so that the viewpoint is from the white matter, looking radially along the main trunk of the descending axon to the soma and dendritic tree of the parent cell. The axon arborisation in the deep layers is drawn in black, with the segments taken for EM analysis indicated in white.

The local cluster around the dendritic tree in the superficial layers has the largest number of boutons, with exponentially declining numbers of boutons in each successive cluster (Binzegger et al. 2004). The main descending axon of the superficial layer pyramidal cells frequently myelinates before it enters the underlying white matter (Martin and Whitteridge 1984a), but the axon collaterals forming the arborisation in layer 5 were unmyelinated, as was the case for the superficial layer clusters (Koestinger et al. 2017). The EM appearance of the boutons was that they were filled with vesicles, contained mitochondria, and they formed asymmetric type 1 synapses with their targets. The labeled axons were clearly distinguished by their dark-staining HRP reaction product in both the light microscope (LM) and the electron microscope (EM). The axons and their synaptic targets were traced through serial ultrathin sections to reconstruct completely the presynaptic bouton and its dendritic target and to be certain of the identity (smooth or spiny) of the postsynaptic dendrites.

**Fig. 2:**
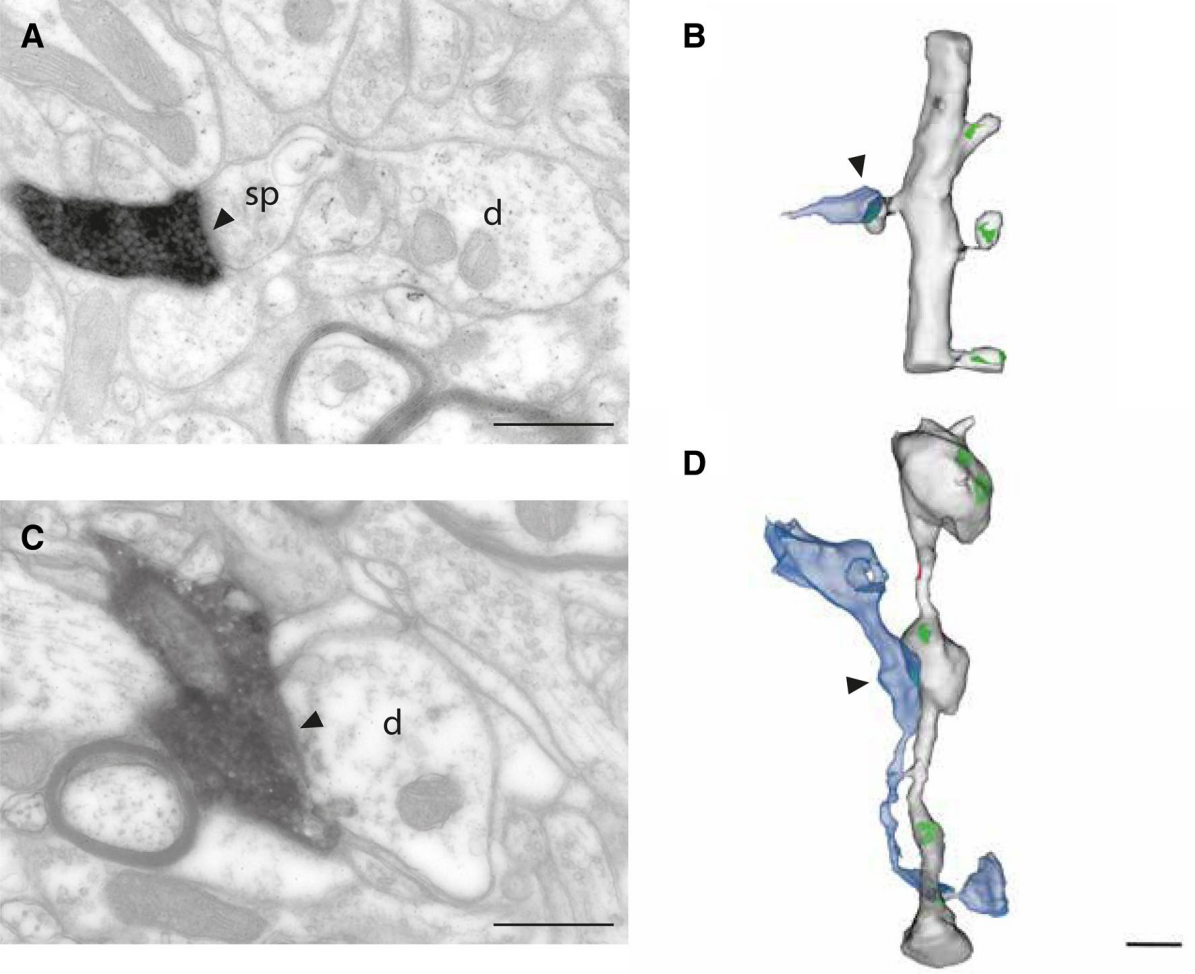
Examples of dendritic targets of labeled axons. Single section through labeled synapses and their corresponding rendered reconstruction from serial EM sections. **a, b** Labeled axon forms synapses with the spiny dendrite (arrowheads). **c, d** Labeled axon forms synapse with the smooth dendrite (arrowheads). Note that the beaded appearance of the dendrite is typical for GABAergic neurons. Scale bars **a, c** 0.5 μm; **b, d** 1 μm

Figure 2 shows the two types of target dendrites: spiny (Fig. 2b) and smooth (Fig. 2d). In Fig. 2a the labeled axon formed an asymmetric synapse (arrowhead) with a spine (sp), whose neck connects it to a dendritic shaft (d). The reconstruction (Fig. 2b) shows that the target dendrite had other spines that formed synapses with unlabeled boutons (labeled axon in blue; asymmetric postsynaptic densities in green). No synapses were formed with the dendritic shaft. By contrast, the dendrite shown in Fig. 2c, d was smooth, so all the synapses were formed with the dendritic shaft. The labeled axon (blue in Fig. 2d, asymmetric postsynaptic densities in green) formed one synapse with a prominent bead on the dendrite. Other unlabeled boutons also formed their synapses mainly with such dendritic beads, which are a feature of smooth neurons in cat V1 (Somogyi et al. 1983; Kisvarday et al. 1985; Ahmed et al. 1997).

**Fig. 3:**
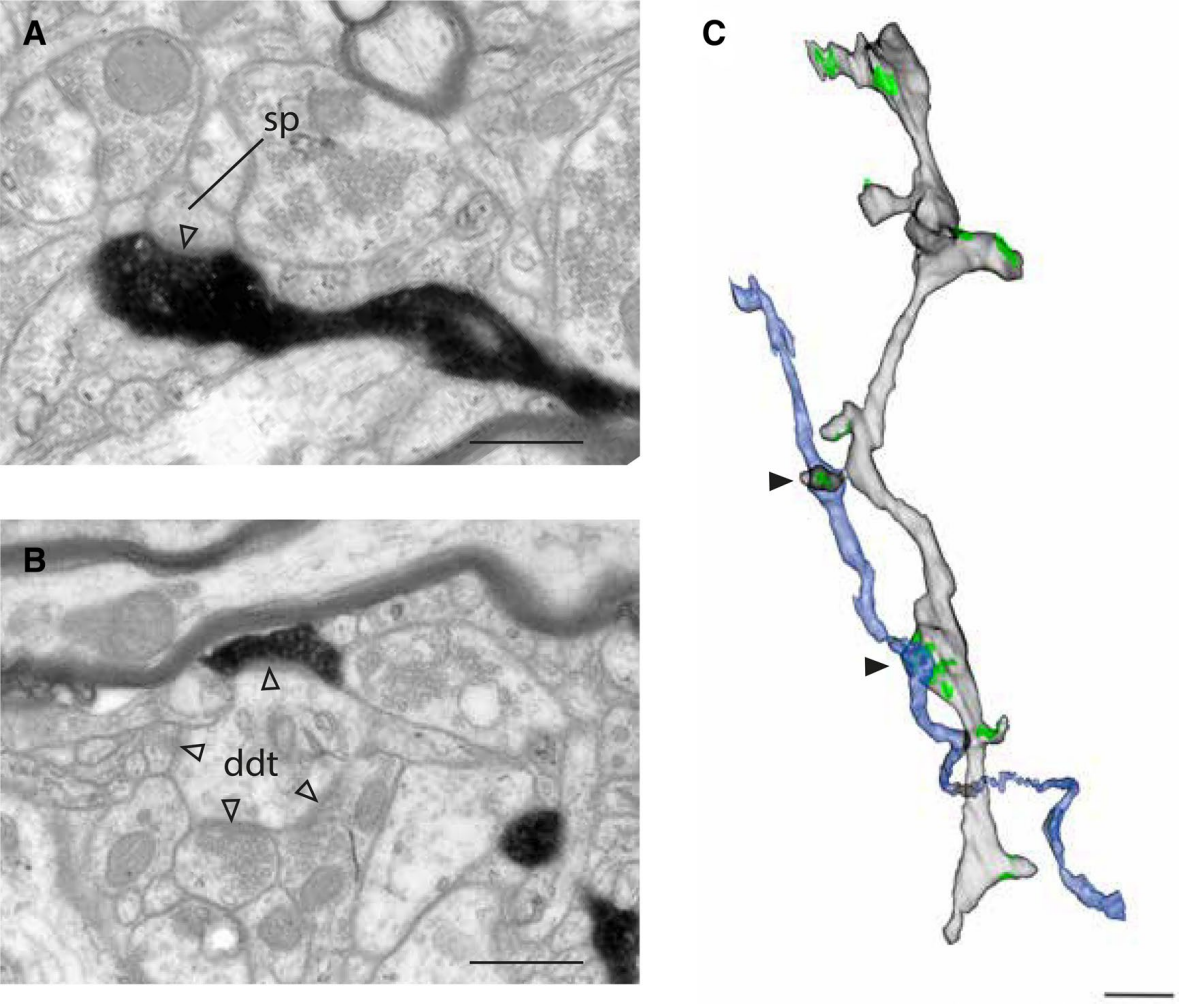
Two synapses formed with the same spiny dendrite. **a** Dendritic spine forms a synapse with the labeled axon. **b** Dendritic shaft forms multiple synaptic inputs. One synapse is with the labeled axon. **c** 3D rendering from serial section EM reconstruction shows the shared target dendrite (light grey) and the axon (blue). Arrowheads indicate the sites of asymmetric synapses (green). Scale bars **a, b** 0.5 μm; **c** 1 μm

The target in Fig. 3a is a spine head and it was traced back to the parent dendritic shaft where another bouton of the labeled axon formed a second synapse about 4 microns from the first (Fig. 3b). Here this region of the dendritic shaft showed features more typical of smooth dendrites: four synapses (arrowheads Fig. 3b), including one with a labeled bouton, formed with a prominent beading of the dendritic shaft (arrowhead at 12 o’clock in Fig. 3b). The rendered reconstruction in Fig. 3c revealed that the same spine-bearing dendrite was the target of both labeled synapses displayed in Fig. 3a, b. If only one or a few sections had been analysed, the target of the second synapse (Fig. 3b) might have been misclassified as being a smooth dendrite.

**Fig. 4:**
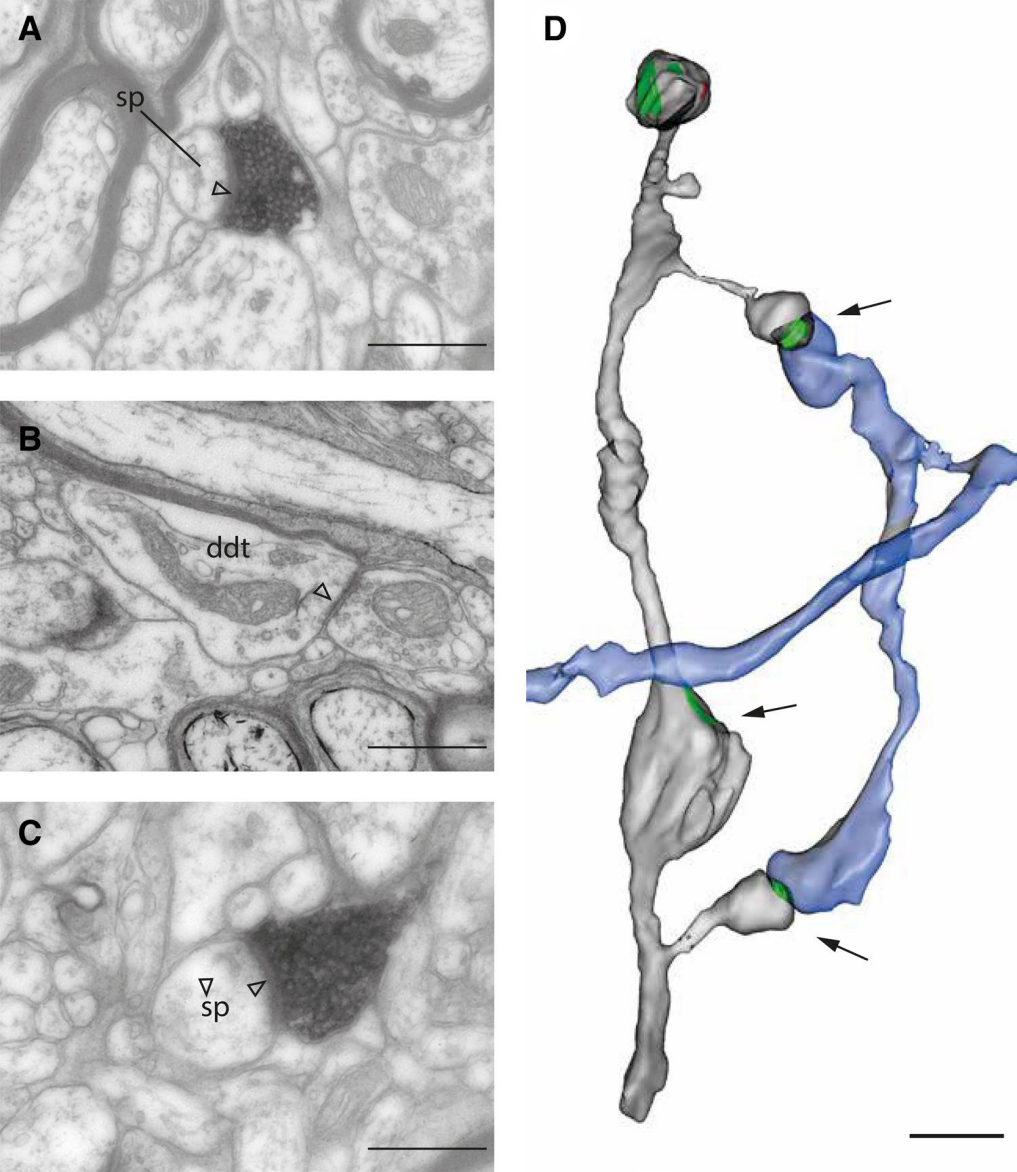
Axon forming a cluster of two synapses with a spiny dendrite. **a**–**c** Electron micrographs of single sections through the synapses formed with a target dendrite. **a, c** Asymmetric synapses formed with spines by the labeled axon. **b** Asymmetric synapse formed with the dendritic shaft by an unlabeled axon. **d** 3D rendering of the serial section reconstruction of the dendritic segment (light grey) and the labeled axon (blue) with asymmetric synapses indicated in green. Black arrows indicate the synapses shown in the electron micrographs of **a**–**c**. Scale bars **a**–**c** 0.5 μm; **d** 1 μm

The beaded shaft of Fig. 3b was not the only such instance: another example of a spiny dendrite with beads on its shaft is shown in Fig. 4. The labeled axon formed two synapses with two spines that connected to the same segment of dendrite (Fig. 4a, c, d). This segment also had a beaded shaft that formed asymmetric synapses with an unlabeled bouton (Fig. 4b). The reconstruction in Fig. 4d shows the spines and beads on the target dendrites, with the axon collateral branching into two terminal boutons that delicately form synapses with the spine heads.

**Fig. 5:**
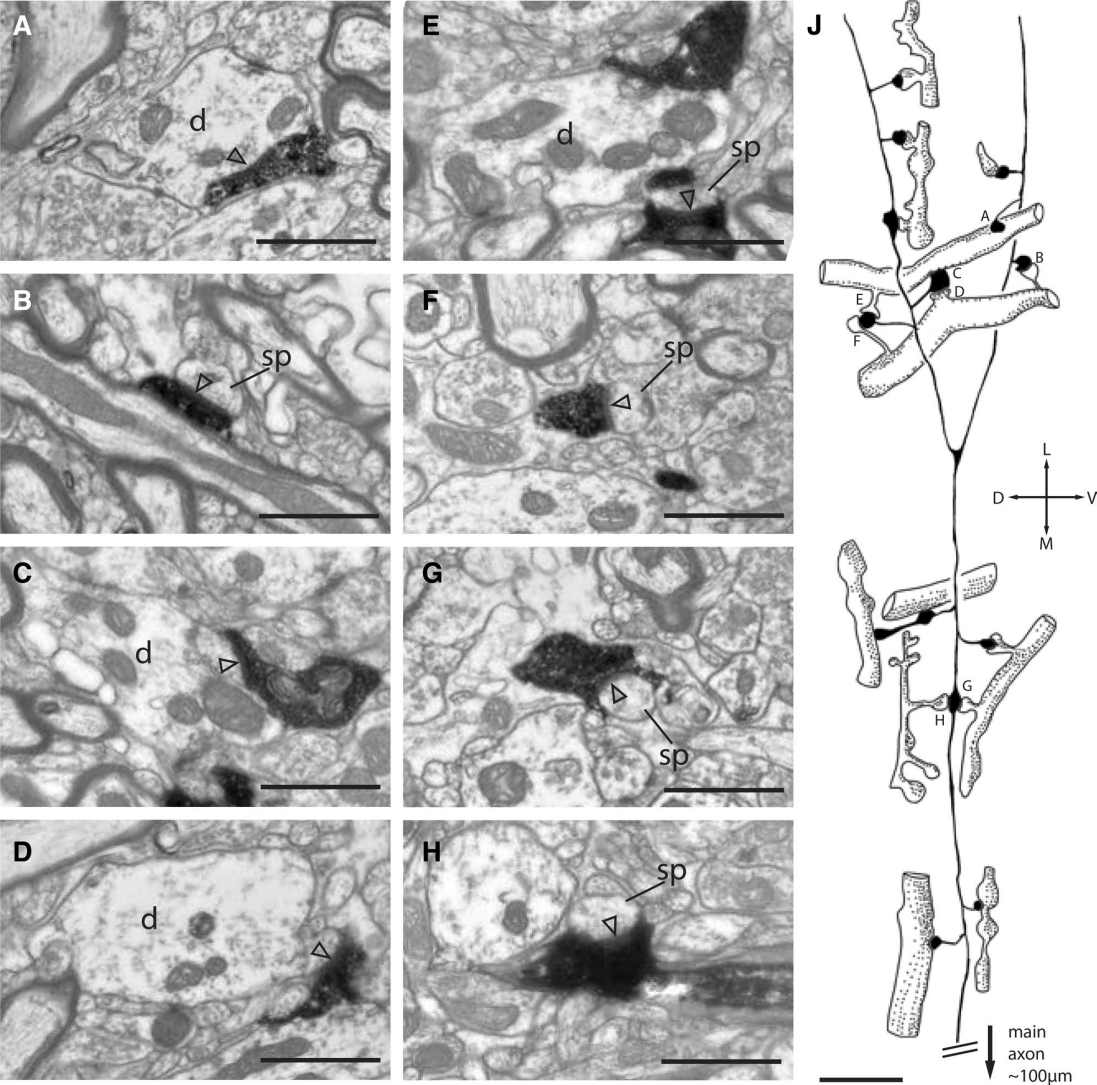
Range of targets that formed one or more synapses with a labeled collateral that branched laterally from the descending main axon. **a, c** Electron micrographs of a labeled axon that forms asymmetric synapses (arrowheads) with the shaft of a spiny dendrite *d*. **b, e, f, h** Labeled axon forms asymmetric synapses (arrowheads) with spine heads (sp), and a stubby spine in **d. j** Sketch showing how the diverse synaptic targets are organized with respect to the labeled collateral. Letters in **j** indicate the boutons corresponding electron micrographs on the left. Orientation of collateral indicated by stereotaxic axes. Scale bar **a**–**h** 1 μm; **j** 5 μm

The trajectory of the axon collaterals gave no hint of the intricacy of their connections to their postsynaptic targets. Figure 5 shows selected micrographs (a–h) and a sketch of one branched collateral (j) that formed synapses with targets that included spines and dendritic shafts of spiny neurons and shafts of smooth neurons. Especially noteworthy are the triple synapses formed with single dendrites (a, c, e and b, d, f) and the fact that two of the boutons involved made double synapses on two separate but parallel spiny dendrites (c, d and e, f), a feature we have not previously encountered (Koestinger et al. 2017). Another bouton also formed two synapses with two spiny dendrites (g, h). The sketch in (j) shows that terminal boutons were much more common than en passant boutons and that they formed synapses with both spines and dendritic shafts. In all, this short segment of the axon collateral formed 14 synapses with 11 different targets.

**Fig. 6:**
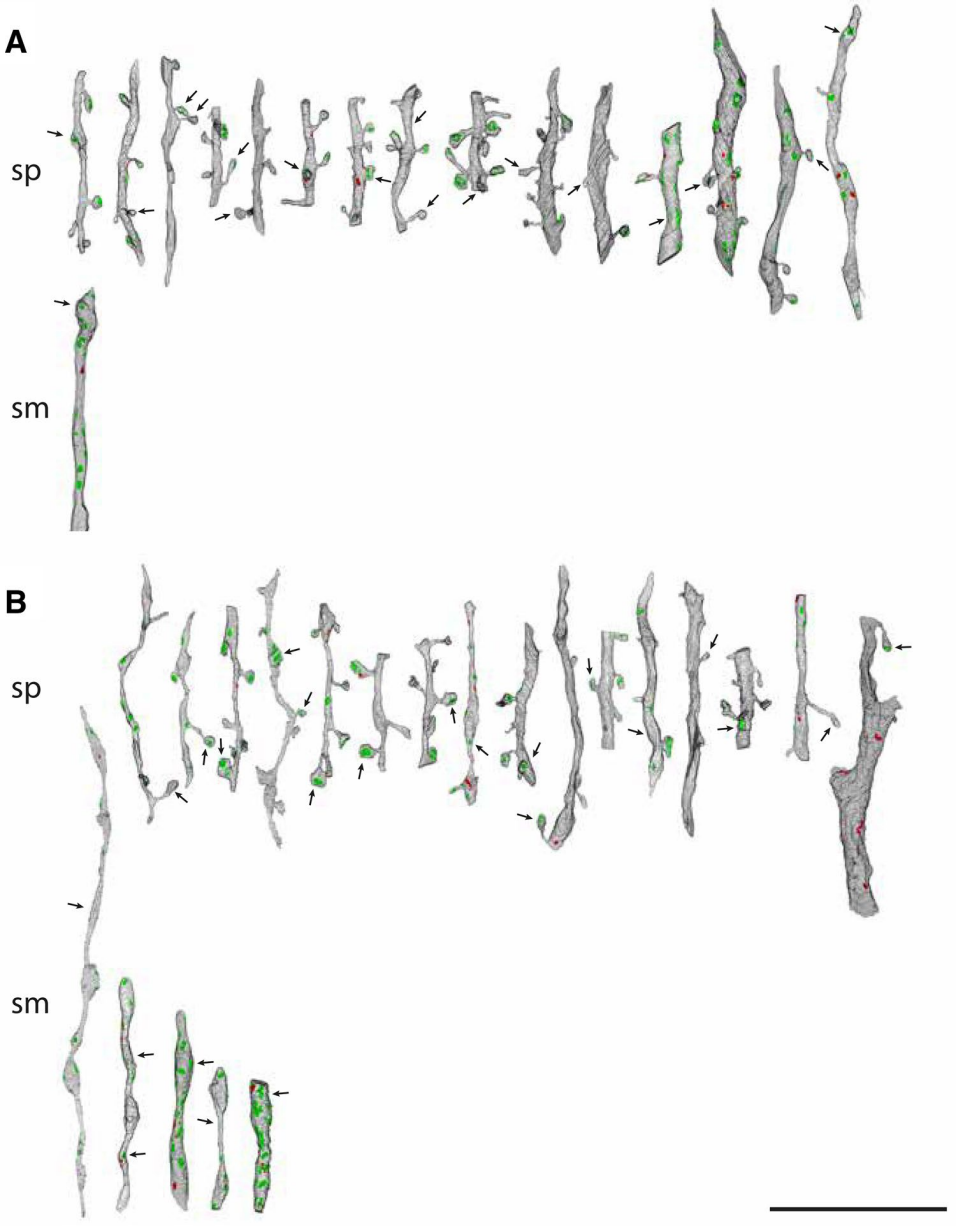

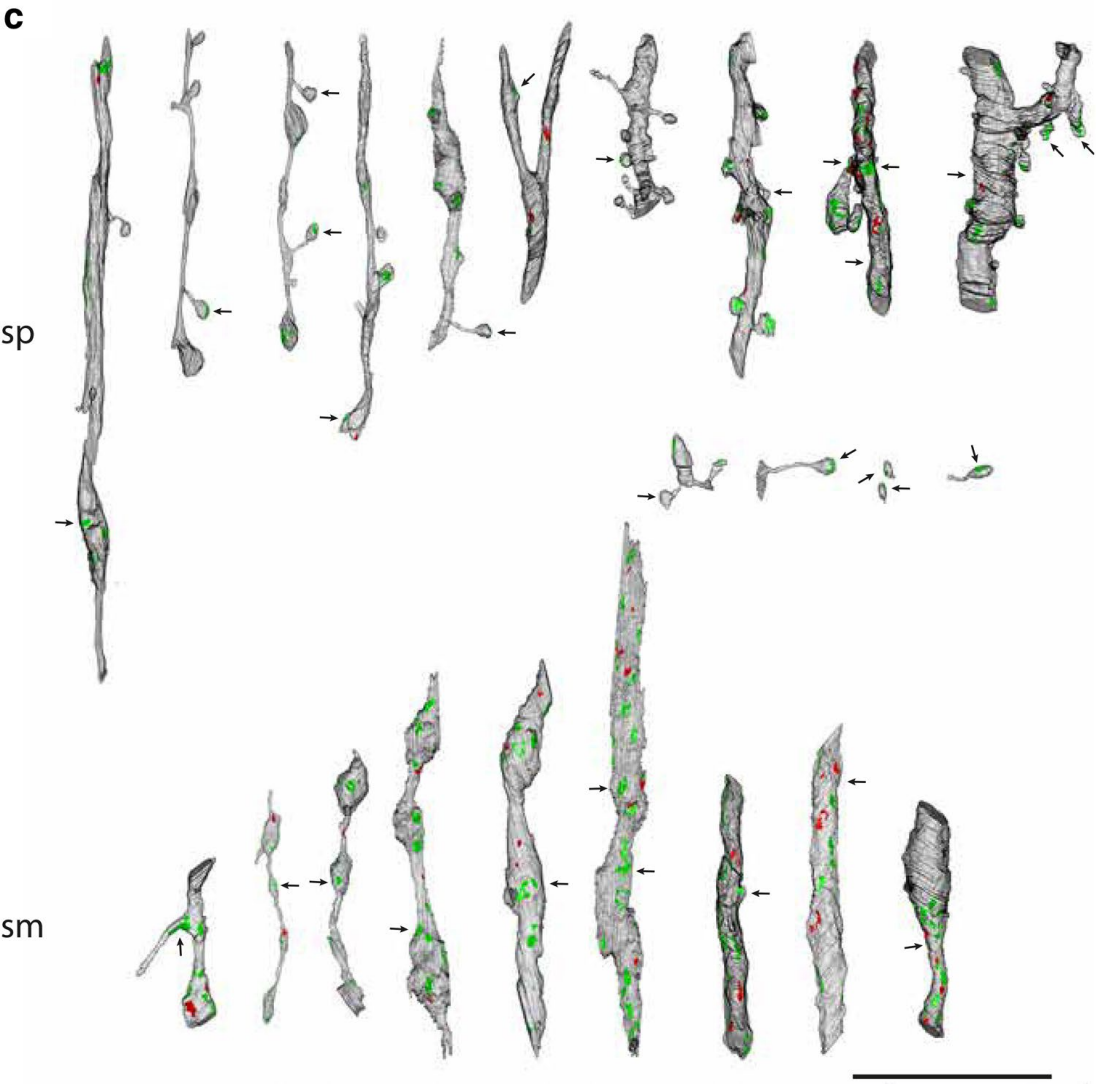
Renderings of reconstructions from serial sections for target dendrites. **a**–**c** Spiny (sp) and smooth (sm) dendritic targets of pyramidal cells 1, 2 and 3, respectively. Postsynaptic densities of asymmetric synapses indicated in green and symmetric synapses in red. Arrows indicate the synapses formed by the labeled axon. Scale bars 10 μm

The full variety of targets of the layer 5 boutons is summarized in Fig. 6. Here all the targets for each of three cells (Fig. 6a–c) have been sorted into spiny and smooth dendrites. The postsynaptic densities of the asymmetric synapses are indicated in green and the symmetric synapses in red. The synapses formed by the labeled axons are arrowed. Spiny dendrites (‘sp’, Fig. 6a–c) were the major postsynaptic target and bore a striking variety of spine morphologies, as the reconstructions show. It is also evident that the spiny dendrites came in a variety of sizes, with thinner dendrites predominating. The thicker dendrites are likely to be from proximal parts of the dendritic trees of pyramidal cells, especially as they formed symmetric synapses, which typically are clustered on the soma and proximal parts of the axon and dendrites (DeFelipe and Farinas 1992).

The other major target was smooth dendrites (‘sm’, Fig. 6a–c). They varied greatly in the prominence of their beads and in the density of synapses formed along their shafts. Most of the synapses on the smooth dendrites were asymmetric. The thicker, synapse-laden segments were likely to be from proximal portions of the dendritic tree, which have a higher synaptic density than more distal portions (Ahmed et al. 1997). As we have observed for layer 4 basket cells (Ahmed et al. 1997), more synapses were formed with the beads than the interbead segments.

**Fig. 7:**
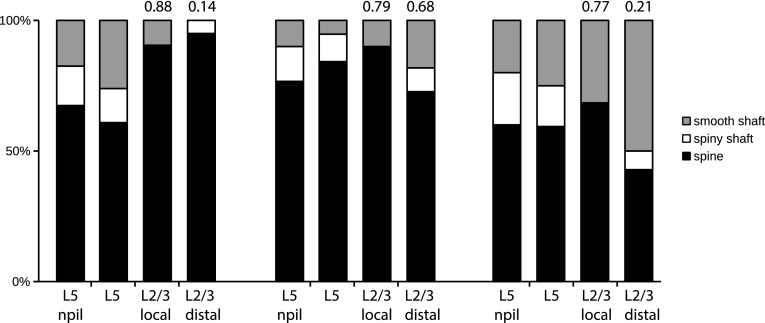
Histograms of proportions of target types (smooth dendrite, spiny dendritic shaft, spine) for cells 1–3, ordered from left to right. The first bar (L5 npil) in each set plots the dissector data of the target types of unlabeled asymmetric synapses sampled from the region immediately around the labeled synaptic boutons. The other bars represent the targets of the axon of one pyramidal cell for the layer 5 cluster (L5) and for the local and distal clusters in the superficial layers (L2/3 local, L2/3 distal). The numbers above the bars indicate the ‘similarity ratio’, which expresses the similarity of the orientation domains occupied by parent dendritic tree to those occupied by its local and distal bouton clusters (see “Methods”)

The summary histograms of Fig. 7 show the proportion of smooth and spiny neurons that were targets, along with the unbiased disector counts of the targets of unlabeled asymmetric synapses in the neuropil in the immediate vicinity of the labeled synapses. A total of 138 synapses were counted for the disectors, 100 of which were asymmetric synapses. For direct comparison, we have also included data for the same cells from samples from the local and distal clusters of boutons formed in the superficial layers, published previously in (Koestinger et al. 2017). The number at the top of the bars is the ‘similarity index’, which express the degree of similarity of the orientation domains occupied by the dendritic tree to the domains occupied by the bouton clusters. Due to light scattering, optical imaging does not permit orientation maps to be made of the deep layers, but the layer 5 clusters we examined probably have similarity indices not very different from that of the local bouton cluster lying radially above them.

We compared the target distributions of the layer 5 boutons with those of the local bouton cluster lying radially above them in the superficial layers. Overall the proportions of target types showed a slightly smaller range of variance in layer 5 (59, 61, 84% of synapses were formed with spiny cells for cells 1, 2, and 3, respectively) compared to the superficial layers (68, 90, 90% of synapses formed with spiny cells for cells I, 2, and 3, respectively). Thus for the same axon, the proportions of the different targets for the local clusters in the superficial layers were not closely matched to the proportions found in their layer 5 clusters. Interestingly, however, there was a close similarity in the proportions of the different target types in the neuropil surrounding the labeled collateral in layer 5, which is what Peters’ Rule of connectivity would predict (Braitenberg and Schüz 1991, 2013).

**Fig. 8:**
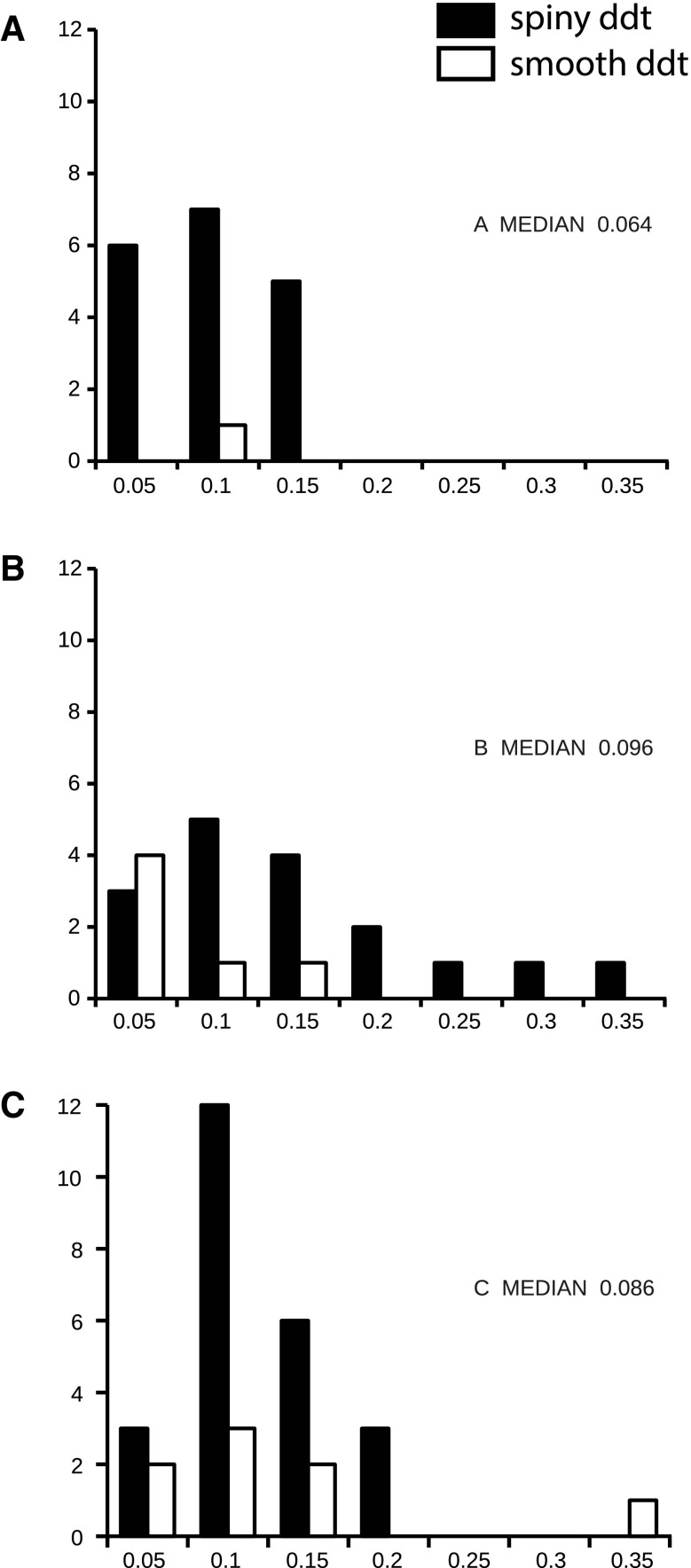
Distribution of areas (μm^2^) of the postsynaptic densities formed by labeled boutons. **a** From Cell 1 (*n* = 19 synapses). **b** From cell 2 (*n* = 23 synapses). **c** From cell 3 (*n* = 32 synapses). Black bars indicate synapses formed with spiny dendritic targets, white bars indicate synapses formed with smooth dendritic targets

We measured the size of the postsynaptic densities (PSD) for all synapses. The distributions for the PSDs of both types of targets are shown separately for each of the three cells in Fig. 8. The long-tailed distribution for one axon is due to the presence of three large synapses, but there was clearly considerable overlap in the PSD sizes for both target types for the three samples.

**Fig. 9:**
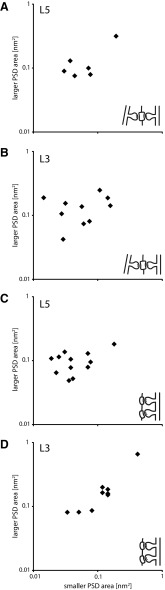
Relationship in sizes of postsynaptic density for double synapses. **a** Relationship of PSD for pairs of synapses that share the same postsynaptic partner in layer 5. **b** Same plot for synapses in layer 3. **c** Relationship in postsynaptic density sizes for boutons that made two synapses, but on two different postsynaptic targets in layer 5. **d** Same plot for synapses in layer 3

In the hippocampus, multiple synapses formed by an axon with the same CA1 pyramidal cell dendrite had PSDs that were much more similar in size than when the axon formed synapses on different postsynaptic dendrites (Bartol et al. 2015). We examined whether such a relation holds for neocortical pyramidal neurons by plotting the PSD sizes for cases where the same collateral formed two or more synapses with the same dendrite (Fig. 9a, b; Note log–log scale). Figure 9 plots the relation of PSD sizes for multisynaptic inputs made by the layer 5 part of the axon (a) and for the layer 3 collaterals of the same axons (b). The plots revealed that PSD sizes varied over a wide range of sizes, without an obvious relation between the two sizes. In layer 5 (Fig. 9a) the mean size for the smaller of the two PSDs was 0.076 μ^2^ (SD 0.061 μ^2^) and the mean size for the larger PSDs of the pair was 0.130 μ^2^ (SD 0.091 μ^2^; Wilcoxon signed rank test *p* = 0.031; *n* = 6). For the layer 3 boutons (Fig. 9b), the mean size of the smaller PSDs of the pair was 0.071 μ^2^ (SD 0.052 μ^2^) and for the larger PSDs of the pair the mean was 0.135 μ^2^ (SD 0.062 μ^2,^; Wilcoxon signed rank test *p* = 0.014; *n* = 12). For comparison we plotted the sizes of the PSDs of individual labeled boutons that formed two synapses with two different postsynaptic structures (Fig. 9c, d). In layer 5 (Fig. 9c) the mean for the smaller of the pair of synapses was 0.053 μ^2^ (SD 0.044 μ^2^) and the mean of the larger of the pair was 0.099 μ^2^ (SD 0.038 μ^2^;Wilcoxon signed rank test *p* = 0.0004; *n* = 12). For the boutons in layer 3 (Fig. 9d), the mean for the smaller of the two PSDs was 0.140 μ^2^ (SD 0.113 μ^2^) and the mean size for the larger of the pair was 0.194 μ^2^ (SD 0.176 μ^2^; Wilcoxon signed rank test *p* = 0.004; *n* = 9). These data indicate that for the axons of the layer 3 pyramidal cells there is no clear relation between PSD sizes for boutons making multiple synapses on the same dendrite in layer 3 or layer 5.

## Discussion

The pyramidal cells of the superficial layers are the major source of excitatory input to neurons lying in the deep layers, which contains the pyramidal cells that provide the sole source of output to subcortical nuclei, such as the thalamus, superior colliculus, pons, and claustrum. Both superficial and deep layer pyramidal cells form reciprocal connections with other cortical areas. The links between superficial and deep layers are a key feature of our canonical circuit for neocortex (Douglas and Martin 1991; Douglas et al. 1989) and provide the means of separating different steps in the cortical computational (Douglas and Martin 2004; Bastos et al. 2012). Here, we studied the critical projection from layer 3 pyramidal cells to layer 5 to determine the spectrum of the postsynaptic targets and compare these patterns with analyses we had previously made of the axonal arborisations in the superficial layers formed by the same cells.

In previous analyses of the layer 3 pyramidal cells we found that the largest bouton cluster (ranked 1, according to the number of boutons) was always located around the region of the dendritic tree itself hence termed the ‘local’ cluster (Binzegger et al. 2007; Martin et al. 2014, 2017). The apical dendrites of the layer 5 pyramidal cells that lie radially beneath the parent cell pass through the local cluster. The bouton cluster in layer 5 that lay radially beneath the soma in layer 3 was the strongest contributor of boutons to layer 5—ranked between second and fifth largest in number of boutons contributed to all clusters (Martin and Whitteridge 1984a; Binzegger et al. 2007). It was from this cluster that we sampled boutons for the present analysis. The major excitatory inputs (> 60%) to the layer 5 pyramidal cells arise from superficial layer pyramidal cells, which connect at two distinct locations—with the apical and the basal dendrites (Binzegger et al. 2004). By contrast, smooth cells receive their portion of input only from the layer 5 arbor because their dendritic trees are largely confined within their layer of origin.

### Postsynaptic targets

In our recent study of the synaptic targets of these same pyramidal cells in the superficial layers, we found a high variance in the proportion of smooth or spiny neurons that were contacted by different pyramidal neurons (Koestinger et al. 2017). This was unexpected, because our previous study of two superficial layer pyramidal cells, which lay in a similar location in the visuotopic map of cat V1, found that pyramidal cells formed consistently about 95% of the targets in both superficial and deep layers (Kisvarday et al. 1987). In contrast, we found high variance in the targets between clusters, both here and in our previous study of the superficial layers (Koestinger et al. 2017). In our recent study of six pyramidal cells, however, we did identify one important source of variance, which was the depth of the parent soma: the closer the soma to layer 2, the fewer synapses its axon formed on average with smooth cells (Koestinger et al. 2017). In the present study, we found that the proportions of smooth vs. spiny targets found in layer 5 bore no relation to those of the local clusters in the superficial layers, and thus no correlation with depth of the parent soma.

Both pyramidal cells in the earlier study of Kisvarday et al. (Kisvarday et al. 1986) lay at the border of layer 3 and 4 and so would be expected to form a high proportion of their synapses with smooth cells if they followed the depth relation reported by Koestinger et al. (2017), but the reverse was the case. It is possible that one species of border pyramidal cell that forms a separate subclass that does not follow the same depth rule of innervation as the pyramidal cells we discovered previously (Koestinger et al. 2017), but since our structural criteria for classifying targets are consistent with those of Kisvarday et al. (1986), the difference between the two studies is real—and still unexplained.

We were curious to discover whether the high variance we had found for the bouton clusters in the superficial layers also applied to the deep layer clusters, and whether the proportion of smooth vs. spiny targets in the deep clusters matched those of the superficial local clusters, as Kisvarday et al. (1986) found for their border pyramids. Binzegger et al. (2004) estimated that 60% of the excitatory synapses found on layer 5 pyramidal cells originated from the pyramidal cells of the superficial layers and consistent with this estimate we found that for both layer 5 and the local cluster in superficial layers, the dominant target was a spine head. The smooth/spiny target proportions of the clusters in the superficial layer and the layer 5 cluster, however, varied greatly. The only consistent feature we identified was that the proportion of smooth vs. spiny targets of the labeled axon was similar to that of the proportions of all targets of asymmetric synapses in the neuropil. Thus, the axons may be simply following Peters’ Rule and be forming synapses with all available targets in the neuropil.

### Identity of target neurons

The morphologies of the target dendrites in layer 5 were very heterogeneous. From their shape, dimensions, orientations, and complement of asymmetric and symmetric synapses, it is likely that apical, oblique and basal dendrites of pyramidal cells were all targets, along with an equally heterogeneous array of smooth dendrites. As reported previously, we found that the pyramidal cells in layer 5 showed the greatest variation of any layer in terms of dendritic and axonal morphology (Martin and Whitteridge 1984a). Quantitative studies in the cat (Gabbott et al. 1987; Hubener et al. 1990) indicate that pyramidal cells in layer 5 vary greatly in their spine densities, even when they project to the same subcortical target, and that individual cells have variations of spine densities from soma to dendritic tip. Some Meynert cells appear to have virtually no spines, despite being the largest pyramidal cells in layer 5 of cat V1 (Gabbott et al. 1987; Hubener et al. 1990). This implies that many asymmetric synapses must form with Meynert cell dendritic shafts, although Gabbott et al. (1987) found that the Meynert cell axons predominantly formed synapses with spines on both the basal and apical dendrites of other Meynert cells, and only a minority of synapses directly on the dendritic shafts. We found shafts of spiny dendrites to be more frequent targets in the layer 5 clusters than in the superficial layer clusters, albeit they were still a minority of targets.

Studies of Golgi-stained material of cat V1 (O’Leary 1941; Lund et al. 1979), in vitro (Thomson et al. 2002; Hubener et al. 1990; Katz 1987; Einstein and Fitzpatrick 1991; Einstein 1996), or in vivo intracellular labeling (Martin and Whitteridge 1984a; Hirsch et al. 1998) indicate there are at least 7 pyramidal cell types in layer 6. All types have an apical dendrite that passes through the layer 5 clusters, and thus all are possible targets. The claustral-projecting cells have an apical dendrite extending to layer with a few oblique branches only in layer 5, whereas the corticothalamic cells have apical dendrites that do not extend further than the top of layer 4, but form a fan of oblique branches within layers 4 and 5 so that their apical dendrite is 50% longer than that of the corticoclaustral cells (Katz 1987; Thomson et al. 2002; Martin and Whitteridge 1984a). Although the apical dendrites of cortico-claustral cells are twice a spiny as those of the cortico-thalamic cells, they offer far fewer synaptic sites in layer 5 because cortico-thalamic cells outnumber the cortico-claustral cells tenfold (Katz 1987).

Smooth neurons—the GABAergic inhibitory cells of the cortex—also formed a minority of the targets in layer 5, but in comparison to the pyramidal cells discussed above, little is known about the dendrites of smooth neurons that inhabit the deep layers of cat V1, even from Golgi studies (Lund et al. 1979; Meyer 1983). Unlike pyramidal cells, which typically are classified according to their layer of origin and features of their apical dendrite, the smooth cells are typically classified on the basis of their axon morphology and not their dendritic morphology, which tends to be similar across types, and thus uninformative as a means of distinguishing between different types.

Very little is known of the species of smooth neurons that populate the deep layers of cat V1. One of the rare studies in cat was of two basket cells located in layer 5 and the border of layer 5 and 6 whose dendrites were largely confined to layers 5 and 6 (Kisvarday et al. 1987). The dendrites branched from 3 to 4 main trunks and, as is typical for smooth cells, the distal dendrites were more beaded than the proximal dendrites. The synaptic input to these basket cells was not examined, but their features described in the LM are consistent with the features we noted in the serial section EM reconstructions, where the thicker smooth dendrites were not beaded. We also observed higher synaptic densities and more symmetric synapses on the unbeaded segments than on the beaded segments. Although the most proximal portions of pyramidal cells are also free of spines and smooth they do not form asymmetric synapses and so are clearly distinguishable from the thick proximal segments of smooth cells. The closest comparison of dendrites of smooth cells is from serial EM reconstructions of basket cell dendrites in layer 4 of cat V1, where the proximal dendrites were relatively thick and covered in synapses, while the beaded segments were distal and synapses were concentrated on the beads (Ahmed et al. 1997).

Interestingly, we could find no evidence that the pyramidal cell collaterals formed more multiple synapses with smooth dendrites than with spiny dendrites, despite the much higher density of asymmetric synapses on smooth dendrites. Like the spiny dendrites, some of the variation in the morphology of the dendrites is because the labeled synapses were likely formed at varying distances from the soma of the target cell. Differences in morphology and synaptic density may also be due to different subtypes of smooth neurons, but since smooth neurons are not classified by dendritic morphology, but by their axonal morphology or by their calcium-binding proteins, we were obviously not able to identify different subtypes. Multiple synapses were found between the labeled axons and both spiny and smooth dendritic targets, but in the majority of cases the labeled axons formed only one synapse with the reconstructed segment of dendrite.

Previously, we made quantitative estimates of the target types of the deep clusters of the superficial layer pyramidal cells based on Peters Rule, which assumes that all dendritic trees passing through layer 5 will be targets of the afferent projections to that layer (Binzegger et al. 2004). The wide variation we observed in the morphology of the targets of the layer 5 cluster is consistent with this assumption. We noted that even single collaterals form synapses with a range of targets. When we identified the targets of unlabeled asymmetric synapses in the neuropil surrounding the labeled collaterals, we found the fractions of smooth and spiny target types were very similar to those of the labeled boutons, implying that the labeled collaterals follow the same local rule of connectivity of all asymmetric synapses in the layer. A similar conclusion was drawn in our previous study of the superficial axon clusters of these same cells (Koestinger et al. 2017). This pattern is in sharp contrast to the superficial layer pyramidal cells in V1 of the mouse, where smooth neurons form a far larger fraction of their targets than would be expected from an analysis of the targets of asymmetric synapses in the surrounding neuropil (Briggman and Bock 2012; Bopp et al. 2014).

As we had previously found for collaterals in the superficial layers (Anderson and Martin 2001), single collaterals formed both types of bouton—terminal or en passant—formed synapses with both spines and dendritic shafts and thereby created complex local geometries involving multiple synapses. It is an interesting question as to whether both pre- and postsynaptic elements actively engage in creating a particular synapse (Nagerl et al. 2007). What the functional consequences are we cannot yet say, but the deployment of terminal boutons rather than en passant boutons to form these synapses was an unusual feature that was not as evident for the collaterals of the same axons in the superficial layers (Koestinger et al. 2017).

### Recurrent circuitry

In our investigations of the intracellular responses to electrical stimulation of the thalamic afferents showed that both excitatory and inhibitory neurons are excited by the same source neurons (Douglas and Martin 1991; Douglas et al. 1989). Our present results provide structural evidence that this is indeed so. Our subsequent structural and modeling studies (Binzegger et al. 2007, 2004) showed that the superficial layer pyramidal cells are far more recurrently connected than the deep layer pyramidal cells, and thus can sustain mutual excitation longer, even in the face of recurrent inhibition. Because the major component of excitatory input to the layer 5 pyramidal cells arises from the superficial layer pyramids, the dynamics of the layer 5 pyramidal cells largely follow those of the superficial layer pyramids (Binzegger et al. 2007). With a single pulse input to the thalamic afferents, however, both excitatory and inhibitory neurons are activated simultaneously to generate the well-described sequence of an EPSP followed by a much longer IPSP. Interestingly, we found that the superficial pyramidal cells had a more prominent EPSP and a slower time-to-peak of the GABAa-mediated IPSP than the deep layer pyramids (Douglas et al. 1989) (Douglas and Martin 1991). We interpreted this as indicating that the GABAa inhibition in the deep layers is stronger than in the superficial layers. Another contributing factor to the voltage response was revealed by our subsequent studies that showed the importance of the recurrent excitation (Binzegger et al. 2004, 2007). Since the recurrent excitation is stronger in superficial than deep layer pyramids stimulation by a brief electrical pulse drives stronger excitation in superficial than in deep layer pyramids, whose recurrent connections are modest (Binzegger et al. 2004, 2007). The pulse drives the feedforward excitatory pathway from the superficial pyramids to the deep layers, but the relatively small number of recurrent connections between the deep layer pyramidal cells (Binzegger et al. 2004) means that the feedfoward inhibition acts against a relatively weaker recurrent excitation than in the superficial layers, and thus achieves a faster time-to-peak (Douglas et al. 1989) (Douglas and Martin 1991).

Intracellular recordings in cat V1 during visual stimulation show that in the majority of cases the orientation preference of the sub-threshold excitatory and inhibitory conductances are similar and slightly more broadly tuned than the suprathreshold spike output (Douglas and Martin 1991; Douglas et al. 1991; Anderson et al. 2000; Borg-Graham et al. 1998). In mouse V1, the spatial extent of excitation and inhibition is apparently similar and balanced (Xu et al. 2016), but in higher mammals the pyramidal axons spread much further than do axons of smooth cells (Binzegger et al. 2007). This spatial difference may contribute to the structural heterogeneity that makes a balanced recurrent circuit hard to achieve (Landau et al. 2016).

### Synapse size

The range of synapse sizes, assessed by the area of the postsynaptic density (PSD) in layer 5 (0.05–0.35 μ^2^) was similar very similar to that of the synapses formed by samples of axon taken in the superficial layers (0.05–0.4 μ^2^); (Koestinger et al. 2017). If, as commonly assumed, the size of the PSD reflects the strength of the synapse, then we would expect to see a similar range of EPSP sizes, assuming all other factors to be equal, like number of synapses, position on the dendritic tree and input conductance of the target neuron. Unfortunately, we are still some way from understanding this correlation in the cat cortex.

In their slice recordings of pairs of layer 5 pyramidal cells in rat S1, Markram et al. (1997) reported that the amplitude of the EPSPs varied 20-fold, but the EPSP amplitudes were only weakly correlated with the number of synapses and their position on the dendritic tree. From simulations they concluded the main source of the amplitude variance was large differences in the probability of transmitter release. Silver et al. (2003) recorded the spiny stellate input to pyramidal cells in rat S1 and concluded there was a 1:1 relationship between the number of synapses seen anatomically and the number of release sites estimated from the physiology. In both studies, however, the pre- and post-synaptic biocytin labeling obscured the details of the synaptic thickenings, so an additional possible contribution to the variance seen in the synaptic physiology—the size of the PSD—could not be assessed. In our tissue only the presynaptic bouton was labeled, so the PSDs were clear and could be reconstructed in 3D. This allowed us to test another hypothesis: that synapse size and, therefore, strength of a synapse, is determined by a Hebb synapse-like mechanism, which is what Bartol et al. (2015) have claimed for hippocampal pyramidal cells.

Bartol et al. (2015) assumed that PSD size correlates with synaptic strength and, therefore, that if an axon makes multiple synapses made on the same pyramidal cell dendrite the synapses should have similar sizes because they experience the same pre- and postsynaptic activity. They reconstructed unlabeled axons and found the postsynaptic densities (PSDs) of such double synapses in rat hippocampus are indeed closely matched in size. Unlike Bartol et al. (2015), however, we know the exact source of the multisynaptic axons. When we compared the PSD size for axons that formed more than one synapse with the same dendrite, we did not find the ‘nearly identical’ size relation reported by Bartol et al. (2015). Instead the two PSDs formed by the same axon on the same dendrite their sizes were poorly correlated. Similarly, when a single bouton formed two synapses on different postsynaptic dendrites we found a similar poor correlation.

### Contribution to receptive field structure

Since the targets of smooth neurons lie in the same orientation domains, the tuning of excitatory and inhibitory inputs is frequently similar (Borg-Graham et al. 1998; Douglas et al. 1991; Anderson et al. 2000; Fournier et al. 2014). In a minority of cases, however, the preferred orientation tuning of the inhibitory conductance is oblique or even orthogonal to the preferred tuning of the excitatory current and spikes (Douglas and Martin 1991; Douglas et al. 1991; Anderson et al. 2000; Borg-Graham et al. 1998; Monier et al. 2003; Martinez et al. 2002). The sharpness of the spike tuning, as well as the magnitude and timing of the excitatory and inhibitory conductances, also varies greatly from cell to cell (Douglas and Martin 1991; Douglas et al. 1991; Anderson et al. 2000; Borg-Graham et al. 1998; Monier et al. 2003; Hirsch et al. 2003). The subthreshold interplay between excitatory and inhibitory inputs determines what precise combinations of inputs actually drive the membrane through spike threshold. Given the heterogeneity in the connections of the patchy excitatory network formed by the superficial layer pyramidal cell that structural and functional studies have revealed (Koestinger et al. 2017; Martin et al. 2017, 2014b; Martin and Schroder 2016; Keller and Martin 2015), it is clear that many different combinations of excitatory and inhibitory inputs lead to the same nett orientation tuning of the spike output.

In the superficial layers of cat V1 the orientation tuning of the spike discharge typically emerges from subthreshold excitatory and inhibitory tuning curves that have the same preferred orientation and Gaussian-shaped profiles (Borg-Graham et al. 1998; Anderson et al. 2000; Monier et al. 2003). In layer 5, however, the preferred orientation for subthreshold excitatory and inhibitory potentials are often quite different and the both the subthreshold tuning curves and the spike tuning curves can be asymmetric in form, i.e. not Gaussian (Monier et al. 2003; Martinez et al. 2002; Fournier et al. 2014). Although the major excitatory input to the layer 5 cells comes from the superficial layer pyramids in the same functional ‘column’, there is clearly a ‘remixing’ of excitatory and inhibitory inputs to the pyramidal cells of layer 5. Monier et al. (2003) argued that this remixing is a means of generating a diversity of responses in the face of complex stimuli, such as feature discontinuities (Sillito et al. 1995; Schmid 2008). Martinez et al. (2002) had made a similar point and suggested further that the information coded in the complex responses of the output cells of layer 5 is likely needed for visually guided behavior in subcortical nuclei, and for the analysis of complex features and motion in higher cortical areas.

## Conclusion

The heterogeneity in the targets we have demonstrated at a single neuron level is consistent with the variety of subthreshold responses recorded intracellularly in vivo. Our observations provide one structural basis for the heterogeneity in the spike responses of neighboring cells when viewing natural stimuli (Gawne et al. 1996; Reich et al. 2001; Weliky et al. 2003; Yen et al. 2007) and why the noise and signal correlations of neighboring cells are so weak, despite their shared location in the same cortical ‘column’ (Martin and Schroder 2016; Cohen and Kohn 2011). But pooling across neurons with different tunings also has distinct advantages: it helps generate invariance in the downstream neurons, it reduces the amplification of shared noise in similarly-tuned neurons, and it provides a means of modulating and augmenting the response of neurons in the processing of complex scenes (Monier et al. 2003; Fournier et al. 2014; Martin and Schroder 2016; Schmidt 2013).

## Methods

The experiments were carried out under licenses granted to KACM by the Kantonales Veterinaeramt of Zurich. Full details of the Methods for optical recording of the orientation maps and the single unit recordings and intracellular injections of horseradish peroxidase, together with cell reconstruction and analysis methods are given in Martin et al. (2014a) and are briefly described here; only the additional methods for the electron microscopy and simulations are described in detail.

### Surgery

Five adult cats of either sex were maintained under general anaesthesia for the duration of the experiment. After craniotomy the cats were given a continuous i.v perfusion of muscle relaxants (gallamine triethiodide, Sigma Aldrich, CH, 13 mg kg^−1^ h^−1^, and (+)-tubocurarine chloride hydrate, Sigma, 1 mg kg^−1^ h^−1^). General anaesthesia was maintained with (30%/70%). Halothane (0.5–1.5%) and continuous i.v. infusion of alphadalone/alphaxalone (Saffan, Glaxo) sufficient to maintain the electroencephalogram (EEG) in a light sleep (spindling) state. EEG, ECG, heart rate, arterial blood pressure, end-tidal CO2 and rectal temperature were monitored continuously during the entire experiment. A thermistor-controlled heating blanket maintained the cat’s rectal temperature at 37°. The eyes protected with gas permeable contact lenses and were refracted to focus on the tangent screen.

### Recording

Glass micropipettes were filled with a 4% solution of Horseradish Peroxidase (HRP, Roche) in 0.05 M Tris and 0.2 M KCl at pH 7.9 and then beveled to impedances between 40 and 88 MΩ (mean 72 MΩ +/− 12). Extracellular receptive fields (RFs) were hand-plotted and classified S or C, simple or complex (Martin and Whitteridge 1984b). In successful attempts to impale the neuron its receptive field was checked to be sure that the extracellular receptive field belonged to the actual neuron and HRP was then iontophoresed into the cell (Martin and Whitteridge 1984a).

### Optical Imaging

For optical imaging a metal chamber (Optical Imaging, Inc) was fixed with dental cement to the skull around the rim of the craniotomy. The camera (CS8310BC, Teli, Japan) was then focused on the surface brain through a macroscope (Ratzlaff and Grinvald 1991) using a wavelength of 546 nm (isobestic for hemoglobin). The camera angle was adjusted so that a larger surface of cortex was in focus and the angle of the macroscope was noted with respect to the stereotaxic planes. A digital image of the cortical surface was taken to record the blood vessel pattern with normal light. Then the illumination wavelength was changed to 700 nm and the camera focused down 450 µm below brain surface (with the 700 nm illumination this corresponded to 600 µm below surface). Image acquisition was synchronized with visual stimulation and five data frames of 600 ms duration were acquired during stimulus presentation (each data frame was the sum of 15 camera frames). Data acquisition was done with an Imager 3001 VSD + setup (Optical Imaging, Inc), and using the software VDAQ (Optical Imaging, Inc).

Visual stimuli consisted of square wave gratings of eight different orientations (0°, 22.5°, 45°, 67.5°, 90°, 112.5°, 135° and 157.5°) with 100% contrast, spatial frequency of 1 cycle·degree-1 and temporal frequency of 1 degree·s-1. During inter-stimulus intervals the next stimulus was presented as stationary. The visual stimuli were displayed to the cat in a random order. All the visual stimuli were programmed in Matlab (MATHworks) and presented using a VSG2/5 graphics card (Cambridge Research).

Single orientation maps (termed as ‘single maps’) were calculated in two ways. Responses from individual orientations (summed activity of 26 to 52 trials) were divided by the summed response to all stimuli (cocktail blank). Alternatively differential maps were calculated in which responses from individual orientations were divided by the orthogonal orientation (Bonhoeffer and Grinvald 1996).

### Alignment of brain sections with the orientation map

Before the perfusion, up to six reference penetrations were made with empty glass pipettes in the stereotaxic coordinate frame. The position of the reference penetrations were noted on the blood vessel pattern and in the stereotaxic coordinates. At the end of the penetration, when the tip of the micropipette was at a depth of 2 mm below the brain surface, the shaft was cut across a few mm above the brain surface and remained in place for perfusion (Phillips et al. 1971). These tracks were identified in histological sections and were used to align the reconstructed neurons with the optical imaging maps.

#### Fixation and histology

At the end of the experiment the cat was euthanized and perfused transcardially with normal 0.9% NaCl solution, followed by a room-temperature solution of 4% paraformaldehyde, 0.3% gluteraldehyde and 15% saturated solution of picric acid in 0.1 M PB pH7.4. After Vibratome sectioning at 80 microns, the HRP was revealed using 3-diaminobenzidine tetrahydrochloride (DAB) with nickel intensification. After assessment by light microscopy (LM) the sections containing labeled neurons were further processed for electron microscopic analyses. These sections were treated with 1% osmium tetroxide in 0.1M PB, dehydrated through alcohols (1% uranyl acetate in the 70% alcohol) and propylene oxide, and flat mounted in Durcupan (Fluka) on glass slides.

### Reconstructions

Neurons were reconstructed in 3-D using a microscope (100x, Olympus BX-51) combined with a motorized stage (MicroBrightField Inc. USA) and the aid of the Neurolucida software (Version 8.0, MicroBrightField Inc. USA). The reconstruction of one neuron took approximately 100 h. While reconstructing the axon, each bouton was tagged with a marker. The borders of cortical layers were determined in tangential sections on the basis of light microscopic characteristics visible in the osmium-treated tissue, such as relative neuron and fiber densities, neural soma size, HRP-filled dendrites, the presence of large pyramidal cells at the border region of layer 3 and 4 and giant pyramidal cells of Meynert in layer 5b.

#### Correlated light and electron microscopy

Serial light micrographs were taken from the osmicated sections at different magnifications and the blood vessel pattern surrounding labeled neurons was reconstructed using TrakEM2 (Cardona et al. 2012). The dendritic arbour and the axon of the neuron of interest were then reconstructed first in 2D using a drawing tube attached to a light microscope, and then in 3D from serial light micrographs using TrakEM2.

For electron microscopy the tissue was serially re-sectioned at 60 nm thickness and collected on Pioloform coated single slot copper grids. The axon of labeled neurons was then found in the ultrathin sections and synapse connectivity between labeled axons and neuropil targets investigated with transmission electron microscopy (TEM). Synapses were classified using conventional criteria and dendrites were classified as smooth or spiny based on the presence of spines and features such as dendritic beads and patterns of innervation. Except for spine-free portions of the proximal dendrites where the symmetric synapses are concentrated, pyramidal neurons form most of their synapses with their spines (Somogyi et al. 1983, 1985; Kisvarday et al. 1985, 1986, 1987; Gabbott et al. 1987; Anderson et al. 1994; Ahmed et al. 1997) The dendrites of smooth neurons necessarily form both asymmetric and symmetric synapses with their dendritic shafts, and have higher densities of both symmetric and asymmetric synapses on the proximal dendrites and on the beads that are found on more distal dendrites (Ahmed et al. 1997).

### Counts of unlabeled synapse targets in the neuropil

We estimated of the percentage of dendritic targets (spines or shafts) using the physical disector method (Sterio 1984). The disector was composed of two serial sections of known thickness (60 nm) separated by one intervening section. Synapses that disappeared from reference to lookup section were counted and the target was classified as dendritic spine or shaft. Both sections were used as reference and lookup doubling the number of disectors per site. Electron micrographs were collected at a resolution of 2.8 nm/pixel with a digital camera (11 mega pixels, Morada, Soft Imaging Systems). The set was sampled from the neuropil surrounding the labeled boutons of recorded neurons. The counts were made of the neuropil around all labeled boutons (74 sites sampled in total) in the layer 5 clusters. The disectors had a size of 5.6 × 3.7 µm. We identified the EM micrograph that contained the first appearance of the postsynaptic density of a synapse formed by a labeled bouton, and then counted all the unlabeled synapses that disappeared in the second-next section, and vice versa. With this procedure we made sure that there is equal sampling for objects with different size (as in the disector method).
